# Loneliness, Lack of Social and Emotional Support, and Mental Health Issues — United States, 2022

**DOI:** 10.15585/mmwr.mm7324a1

**Published:** 2024-06-20

**Authors:** Katherine V. Bruss, Puja Seth, Guixiang Zhao

**Affiliations:** 1 Division of Population Health, National Center for Chronic Disease Prevention and Health Promotion, CDC.

**Summary**

**What is already known about this topic?**

Loneliness and lack of social connection are widespread and pose a threat to mental and physical health.

**What is added by this report?**

In 2022, the prevalence of feeling lonely always, usually, or sometimes among adults in 26 U.S. states was highest for bisexual (56.7%) and transgender persons (range = 56.4%–63.9%); these groups also reported the highest prevalence of stress, frequent mental distress, and history of depression (range = 34.3%–67.2%). Prevalence of lack of social and emotional support was elevated among transgender adults.

**What are the implications for public health practice?**

Addressing the threat to mental health among sexual and gender minority groups should include consideration of loneliness and lack of social and emotional support.

## Abstract

Loneliness and lack of social connection are widespread and negatively affect physical and mental health and well-being. Data are limited for persons disproportionately affected by social disconnection, especially those who do not identify as heterosexual and cisgender. Using data from the 2022 Behavioral Risk Factor Surveillance System in 26 U.S. states, CDC examined associations of loneliness and lack of social and emotional support to mental health variables. Prevalence estimates for the mental health variables were significantly higher among adults who reported loneliness and lack of social and emotional support than among those adults who did not. The prevalence of loneliness was highest among respondents who identified as bisexual (56.7%) and transgender (range = 56.4%–63.9%). Prevalence of lack of social and emotional support was highest among those who identified as transgender female (44.8%), transgender gender nonconforming (41.4%), and those with household income below $25,000 (39.8%). Prevalences of stress, frequent mental distress, and history of depression were highest among bisexual (34.3%–54.4%) and transgender adults (36.1%–67.2%). Addressing the threat to mental health among sexual and gender minority groups should include consideration of loneliness and lack of social and emotional support. Providing access to health services that are affirming for sexual and gender minority groups and collecting data to address health inequities might help improve the delivery of culturally competent care.

## Introduction

Social connection is a social determinant of health associated with significant health benefits (*1*). Social connection reflects the degree to which persons have and perceive a desired number, quality, and diversity of relationships that create a sense of belonging, and of being cared for, valued, and supported. Loneliness and isolation are indicators of social disconnection that can lead to poor mental and physical health outcomes, including increased risk for heart disease, stroke, dementia, type 2 diabetes, depression, anxiety, and premature mortality (*1*–*3*). Although these risks are well documented, a more comprehensive understanding of the impact of loneliness and lack of social and emotional support on mental health–related outcomes is needed, particularly among persons experiencing the most social disconnection, such as those who do not identify as heterosexual and cisgender. Sexual and gender minority (SGM) data are often not collected in research, resulting in a lack of data on and evidence-based interventions for loneliness and lack of social and emotional support among these groups (*4**,**5*). The objectives of this study were to assess the association between social connection and mental health among U.S. adults and to determine the prevalence of loneliness, lack of social and emotional support, and mental health issues by demographic characteristics, including sexual orientation and gender identity, to guide prevention and intervention efforts.

## Methods

### Data Source and Definitions

This study examined the association between loneliness and lack of social and emotional support, which are indicators of social disconnection, and mental health measures that included stress, frequent mental distress, and history of depression (Box) and assessed prevalence of these factors by demographic characteristics, including sexual orientation and gender identity, using data from the 2022 Behavioral Risk Factor Surveillance System (BRFSS).* BRFSS is a state-based landline and cellular telephone survey of noninstitutionalized U.S. civilian residents aged ≥18 years; the survey collects data on health-related risk behaviors, chronic diseases and conditions, health care access, and use of preventive services.

BOX **Social connection and mental health variables — Behavioral Risk Factor Surveillance System, United States, 2022**

**Loneliness**

• Defined as a response of “always/usually/sometimes” to the question, “How often do you feel socially isolated from others? Is it always, usually, sometimes, rarely, never, don’t know/not sure, refused?”

• The Office of the Surgeon General defines loneliness as a subjective distressing experience that results from perceived isolation or inadequate meaningful connections, where inadequate refers to the discrepancy or unmet need between a person’s preferred and actual experience.

**Lack of social and emotional support**

• Defined as a response of “sometimes/rarely/never” to the question, “How often do you get the social and emotional support that you need? Is that always, usually, sometimes, rarely, never, don’t know/not sure, refused?”

**Stress**

• Defined as a response of “always/usually” to the question, “Stress means a situation in which a person feels tense, restless, nervous or anxious, or is unable to sleep at night because their mind is troubled all the time. Within the last 30 days, how often have you felt this kind of stress? Was it always, usually, sometimes, rarely, never, don’t know/not sure, refused?”

**Frequent mental distress**

• Defined as a response of “14” or more days to the question, “Now thinking about your mental health, which includes stress, depression, and problems with emotions, for how many days during the past 30 days was your mental health not good?”

**History of depression**

• Defined as a response of “Yes” to the question, “Has a doctor, nurse, or other health professional ever told you had a depressive disorder (including depression, major depression, dysthymia, or minor depression)?”

### Study Participants

Twenty-six states, including 236,866 participants, used both the BRFSS Social Determinants and Health Equity module and the BRFSS Sexual Orientation and Gender Identity module. Participants who responded “don’t know/not sure,” refused to answer, or had missing responses for demographic variables including age, sex, race and ethnicity, education, marital status, and the number of adults and children living in household were excluded, resulting in an analytic sample of 218,915 participants. Participants with missing information for household income, sexual orientation, and gender identity were included as an unknown group because of high proportions (15.5%–20.1%) of missing responses. Missing responses for social connection and mental health measures were further excluded from respective analyses, ranging from 0.5% for history of depression to 15.2% for stress. Details of the 2022 BRFSS Social Determinants and Health Equity module are described elsewhere (*6*).

### Data Analysis

Adjusted prevalence ratios of loneliness and lack of social and emotional support with mental health variables were estimated using log-linear regression analyses with robust variance estimator and adjustment for demographic characteristics. Weighted prevalence estimates for loneliness, lack of social and emotional support, and mental health variables with 95% CIs were calculated, stratified by demographic variables. Statistical significance was determined based on whether there was an overlap between 95% CIs for any two estimates. Analyses were conducted using SAS-callable SUDAAN (version 11.0.3; RTI International) to account for the complex survey design, following the procedures listed in the yearly complex sampling weights and module analysis guidelines.^†^ This activity was reviewed by CDC, deemed not research, and was conducted consistent with applicable federal law and CDC policy.^§^

## Results

### Association of Social Connection with Mental Health Variables

Prevalence estimates for the three mental health measures were significantly higher among adults who reported loneliness and lack of social and emotional support than among those who did not (Table 1). After adjustment for demographic characteristics and sexual orientation and gender identity variables, the adjusted prevalence ratios for stress, frequent mental distress (FMD), and history of depression (depression) among adults who reported loneliness were 3.61, 3.05, and 2.38 times as high, respectively, as were those among adults who did not. Compared with adjusted prevalence ratios among adults who did not report lack of social and emotional support, adjusted prevalence ratios for mental health outcomes were elevated among those who did (3.0 [stress], 2.6 [FMD], and 1.8 [depression]).

**TABLE 1:** Association between social connection and mental health variables — Behavioral Risk Factor Surveillance System, United States, 2022

Social connection variables	Mental health variables					
	Stress		Frequent mental distress		History of depression	
	% (95% CI)	APR* (95% CI)	% (95% CI)	APR* (95% CI)	% (95% CI)	APR* (95% CI)
**Loneliness**						
No	6.4 (6.2–6.7)	Ref	8.4 (8.0–8.7)	Ref	13.8 (13.4–14.1)	Ref
Yes	29.6 (28.7–30.4)	3.6 (3.4–3.8)	32.2 (31.3–33.1)	3.0 (2.9–3.2)	38.4 (37.5–39.2)	2.4 (2.3–2.5)
**Lack of social and emotional support**						
No	8.6 (8.3–8.9)	Ref	10.6 (10.3–11.0)	Ref	17.6 (17.3–18.0)	Ref
Yes	30.9 (29.9–31.9)	3.0 (2.9–3.2)	33.2 (32.1–34.2)	2.6 (2.5–2.7)	34.8 (33.9–35.8)	1.8 (1.8–1.9)

**Abbreviations**: APR = adjusted prevalence ratio; Ref = referent group.

* Adjusted for age, sex, race and ethnicity, education, marital status, household income, number of persons living in household, sexual orientation, and gender identity.

### Weighted Prevalence Estimates for Social Connection Measures

Overall prevalence estimates were 32.1% for loneliness and 24.1% for lack of social and emotional support (Table 2). Within the corresponding demographic categories, prevalences of loneliness and lack of social and emotional support were respectively highest among those aged 18–34 years (43.3% and 29.7%), those with less than a high school education (41.1% and 36.3%), those who never married (45.9% and 34.7%), and those with household income below $25,000 (47.9% and 39.8%); prevalences were lowest among non-Hispanic White adults (29.6% and 20.1%) and those who had two adults living in a household (27.4% and 19.1%). Loneliness was significantly more common among women than among men (33.5% versus 30.7%), whereas lack of social and emotional support was more common among men than among women (22.3% versus 26.1%).

**TABLE 2:** Social connection and mental health variables, by demographic characteristics — Behavioral Risk Factor Surveillance System, United States,* 2022

Characteristic	No. of respondents	% (weighted)	Social connection measure % (95% CI)		Mental health measure % (95% CI)		
			Loneliness	Lack of social and emotional support	Stress	Frequent mental distress	History of depression
**Overall**	**218,915^†^**	**100.0**	**32.1 (31.7–32.6)**	**24.1 (23.6–24.5)**	**13.9 (13.6–14.3)**	**16.0 (15.6–16.3)**	**21.3 (20.9–21.6)**
**Age group, yrs**							
18–34	36,479	29.2	43.3 (42.2–44.3)	29.7 (28.7–30.8)	21.6 (20.7–22.5)	23.2 (22.4–24.1)	26.4 (25.6–27.2)
35–49	44,211	23.3	31.9 (31.0–32.9)	24.6 (23.7–25.5)	16.0 (15.3–16.7)	16.3 (15.6–17.0)	22.0 (21.3–22.7)
50–64	59,085	24.8	27.8 (27.0–28.7)	22.0 (21.2–22.8)	11.5 (11.0–12.1)	13.4 (12.9–14.0)	20.0 (19.3–20.6)
≥65	79,140	22.7	23.8 (23.1–24.5)	19.1 (18.4–19.8)	5.4 (5.0–5.9)	9.0 (8.6–9.5)	15.3 (14.8–15.8)
**Sex**							
Men	103,005	48.4	30.7 (30.0–31.3)	26.1 (25.4–26.7)	11.6 (11.2–12.1)	12.9 (12.5–13.3)	15.1 (14.6–15.5)
Women	115,910	51.6	33.5 (32.8–34.1)	22.3 (21.7–22.9)	16.0 (15.5–16.5)	18.8 (18.3–19.4)	27.0 (26.5–27.6)
**Race and ethnicity^§^**							
AI/AN	3,410	1.3	37.9 (31.7–44.5)	28.1 (24.2–32.3)	17.2 (14.3–20.6)	20.9 (17.7–24.5)	25.4 (22.2–28.9)
Asian	5,492	5.1	32.0 (29.1–35.0)	31.6 (28.8–34.4)	9.5 (7.4–12.1)	10.8 (9.1–12.7)	10.7 (9.2–12.4)
Black or African American	15,306	12.2	36.4 (34.7–38.0)	33.6 (32.0–35.2)	13.5 (12.4–14.7)	16.9 (15.9–18.1)	16.2 (15.2–17.2)
Native Hawaiian or Pacific Islander	451	0.4	38.3 (29.2–48.4)	34.0 (25.6–43.6)	22.5 (15.3–31.7)	22.2 (15.8–30.4)	19.7 (14.2–26.8)
White	170,844	62.4	29.6 (29.1–30.1)	20.1 (19.7–20.6)	13.3 (12.9–13.7)	15.6 (15.2–16.0)	23.2 (22.8–23.6)
Hispanic or Latino	19,070	15.4	37.5 (35.9–39.1)	29.9 (28.4–31.4)	16.0 (14.8–17.2)	15.9 (14.8–17.0)	18.1 (17.1–19.2)
Multiracial	4,342	3.2	40.1 (37.2–43.0)	28.6 (26.0–31.3)	22.9 (20.3–25.7)	25.0 (22.6–27.6)	32.3 (29.8–35.0)
**Education**							
Less than high school diploma	11,912	10.7	41.1 (39.1–43.1)	36.3 (34.4–38.3)	19.1 (17.5–20.8)	20.4 (18.9–21.9)	22.4 (21.0–23.8)
High school diploma or GED	52,899	28.0	34.7 (33.8–35.7)	27.5 (26.6–28.4)	15.1 (14.4–15.8)	18.3 (17.6–19.0)	21.0 (20.3–21.7)
Some college	59,061	30.4	33.0 (32.2–33.9)	24.6 (23.8–25.4)	15.4 (14.8–16.1)	17.7 (17.1–18.3)	24.4 (23.8–25.1)
College and above	95,043	31.0	26.0 (25.4–26.6)	16.5 (16.0–17.1)	9.7 (9.3–10.1)	10.7 (10.3–11.2)	18.0 (17.5–18.4)
**Marital status**							
Married or unmarried couple	125,349	56.2	24.9 (24.3–25.5)	17.2 (16.7–17.7)	10.5 (10.1–10.9)	11.9 (11.5–12.3)	17.3 (16.8–17.7)
Previously married	55,401	19.4	36.3 (35.3–37.2)	31.1 (30.2–32.1)	15.6 (14.8–16.3)	18.9 (18.2–19.7)	26.1 (25.3–26.8)
Never married	38,165	24.5	45.9 (44.8–47.1)	34.7 (33.6–35.8)	20.6 (19.6–21.6)	23.0 (22.2–23.9)	26.7 (25.8–27.6)
**Household income,**^¶^ **$**							
<25,000	25,556	12.5	47.9 (46.3–49.4)	39.8 (38.3–41.3)	24.1 (22.8–25.4)	27.2 (26.0–28.5)	32.0 (30.7–33.3)
25,000–49,999	43,404	19.8	36.2 (35.2–37.2)	29.6 (28.6–30.7)	16.3 (15.5–17.2)	19.8 (19.0–20.6)	24.1 (23.3–24.9)
50,000–74,999	30,624	12.8	30.4 (29.3–31.6)	22.4 (21.3–23.5)	12.7 (11.9–13.6)	15.3 (14.5–16.2)	21.4 (20.4–22.3)
75,000–99,999	25,783	10.9	27.0 (25.8–28.3)	18.5 (17.4–19.6)	11.4 (10.5–12.5)	12.4 (11.6–13.3)	19.3 (18.3–20.3)
100,000–149,999	27,594	11.9	23.6 (22.5–24.8)	14.6 (13.7–15.5)	9.6 (8.9–10.3)	10.5 (9.8–11.3)	17.6 (16.7–18.5)
≥150,000	25,533	12.0	21.2 (20.2–22.3)	13.8 (12.8–14.7)	8.2 (7.5–8.8)	8.7 (8.0–9.4)	14.2 (13.4–14.9)
Unknown	40,421	20.1	34.7 (33.5–36.0)	25.6 (24.5–26.8)	13.7 (12.7–14.7)	15.3 (14.5–16.1)	19.3 (18.5–20.1)
**No. of children living in household**							
0	161,933	66.3	31.9 (31.4–32.5)	23.5 (23.0–24.0)	12.9 (12.5–13.4)	15.7 (15.3–16.1)	21.5 (21.1–22.0)
1	23,811	13.8	33.5 (32.3–34.8)	25.5 (24.3–26.7)	16.8 (15.8–17.8)	16.9 (16.0–17.9)	21.6 (20.7–22.6)
2	20,125	11.7	31.6 (30.1–33.0)	24.6 (23.3–26.0)	15.1 (14.0–16.3)	15.4 (14.4–16.5)	19.9 (18.9–21.0)
3	8,565	5.2	31.2 (28.9–33.6)	24.6 (22.5–26.8)	14.6 (13.1–16.2)	15.8 (14.4–17.3)	20.6 (19.0–22.4)
4	2,896	1.9	34.5 (30.5–38.7)	26.8 (23.7–30.3)	17.4 (14.7–20.4)	18.9 (16.3–21.7)	19.8 (17.4–22.5)
≥5	1,585	1.1	32.4 (26.8–38.4)	30.5 (25.2–36.5)	17.0 (13.2–21.8)	21.6 (17.2–26.7)	19.8 (16.0–24.2)
**No. of adults living in household**							
1	62,140	22.8	38.7 (37.8–39.6)	31.6 (30.7–32.5)	15.4 (14.7–16.1)	18.4 (17.7–19.1)	24.3 (23.5–25.0)
2	109,832	47.8	27.4 (26.8–28.0)	19.1 (18.5–19.6)	11.6 (11.2–12.1)	13.6 (13.1–14.0)	19.4 (18.9–19.8)
3	28,544	16.6	34.3 (33.0–35.6)	25.6 (24.4–26.9)	16.2 (15.2–17.3)	17.4 (16.5–18.3)	22.3 (21.3–23.3)
4	12,687	8.7	35.1 (33.1–37.0)	27.0 (25.2–28.9)	15.5 (14.1–17.0)	17.9 (16.4–19.5)	21.3 (19.9–22.7)
≥5	5,712	4.1	37.6 (34.5–40.8)	30.0 (27.4–32.8)	19.9 (17.1–23.1)	20.8 (18.9–23.0)	22.3 (20.2–24.5)
Sexual orientation^¶,^**							
Gay	2,195	1.0	41.2 (37.5–45.1)	29.0 (25.5–32.7)	18.3 (15.4–21.5)	20.8 (17.9–24.1)	34.0 (30.5–37.7)
Lesbian	1,784	0.8	44.8 (40.5–49.2)	26.0 (22.2–30.1)	27.7 (23.9–31.9)	30.5 (26.7–34.6)	43.2 (39.1–47.5)
Bisexual	6,295	3.8	56.7 (54.1–59.3)	36.5 (33.8–39.3)	34.3 (31.7–37.0)	40.5 (37.9–43.1)	54.4 (51.8–56.9)
Straight	179,201	75.5	30.3 (29.8–30.8)	22.8 (22.4–23.2)	12.6 (12.2–12.9)	14.2 (13.9–14.6)	19.4 (19.0–19.8)
Something else	3,390	1.8	50.7 (47.2–54.2)	39.3 (36.0–42.6)	28.1 (25.4–31.1)	33.0 (30.1–36.1)	39.3 (36.3–42.5)
Unknown	26,050	17.2	31.0 (29.4–32.5)	26.0 (24.6–27.4)	11.4 (10.4–12.4)	15.5 (14.7–16.4)	18.5 (17.7–19.4)
Gender identity^¶,^**							
Transgender female	372	0.2	56.4 (42.4–69.5)	44.8 (32.0–58.3)	36.1 (25.9–47.6)	37.2 (27.3–48.4)	47.4 (35.9–59.3)
Transgender male	408	0.2	62.6 (54.6–70.0)	34.4 (26.7–43.1)	36.4 (28.8–44.8)	39.8 (32.5–47.6)	48.8 (41.0–56.8)
Transgender gender nonconforming	455	0.3	63.9 (55.9–71.3)	41.4 (34.2–49.0)	37.8 (30.9–45.1)	51.8 (44.0–59.5)	67.2 (59.4–74.1)
Cisgender	194,978	83.8	32.1 (31.6–32.5)	23.8 (23.4–24.3)	13.9 (13.5–14.2)	15.7 (15.4–16.1)	21.4 (21.0–21.7)
Unknown	22,702	15.5	29.7 (28.2–31.3)	25.4 (23.9–26.9)	11.7 (10.7–12.9)	16.1 (15.2–17.0)	19.2 (18.3–20.1)

**Abbreviations**: AI/AN = American Indian or Alaska Native; GED = general education development certificate; SOGI = sexual orientation and gender identity.

* The following states used both the Social Determinants and Health Equity and SOGI modules: Alaska, Connecticut, Delaware, Georgia, Indiana, Iowa, Kansas, Maryland, Massachusetts, Michigan, Minnesota, Missouri, Montana, Nevada, New Jersey, New Mexico, North Carolina, Ohio, Oklahoma, Rhode Island, Texas, Utah, Vermont, Washington, West Virginia, and Wisconsin.

^†^ Analytic sample size is 218,915 after excluding those who responded “don’t know/not sure,” refused to answer, or had missing responses for demographic variables (except for those with unknown income, sexual orientation, and gender identity status).

^§^ Persons of Hispanic or Latino (Hispanic) origin might be of any race but are categorized as Hispanic; all racial groups are non-Hispanic. Other categories reported as a single race or as multiracial when more than one race was reported.

^¶^ Because of high proportions of missing responses for household income, sexual orientation, and gender identity, data for these three variables are combined into an "Unknown” group.

** Information on the SOGI module is available at https://www.cdc.gov/brfss/data_documentation/pdf/BRFSS-SOGI-Stat-Brief-508.pdf, and on terminology at https://www.cdc.gov/healthyyouth/terminology/sexual-and-gender-identity-terms.htm.

The prevalence of loneliness was significantly higher among adults who identified as gay (41.2%), lesbian (44.8%), bisexual (56.7%), or something other than gay, lesbian, bisexual, or straight (50.7%), than among those who identified as straight (30.3%). Loneliness was significantly higher among adults who were transgender female (56.4%), transgender male (62.6%), or transgender gender nonconforming (63.9%), than among those who were cisgender (32.1%). Lack of social and emotional support was significantly more prevalent among adults who identified as gay (29.0%), bisexual (36.5%) and something other than gay, lesbian, bisexual, or straight (39.3%), than among those who identified as straight (22.8%); prevalence among adults who were transgender female (44.8%), transgender male (34.4%), or transgender gender nonconforming (41.4%) was significantly greater than among those who were cisgender (23.8%).

### Weighted Prevalence Estimates for Mental Health Measures

Overall prevalence estimates were 13.9% for stress, 16.0% for FMD, and 21.3% for depression (Table 2). Within the corresponding demographic categories, prevalences of mental health measures were the highest among those aged 18–34 years (21.6% [stress], 23.2% [FMD], 26.4% [depression]), females (16.0% [stress], 18.8% [FMD], and 27.0% [depression]), and those with less than a high school education (19.1% [stress] and 20.4% [FMD]). Prevalence of depression by education was the highest among those with some college (24.4%). Prevalences were also highest among those never married (20.6% [stress] and 23.0% [FMD]) and those with household income below $25,000 (24.1% [stress], 27.2% [FMD], and 32.0% [depression]). Prevalences were lowest among non-Hispanic Asian persons (9.5% [stress], 10.8% [FMD], and 10.7% [depression]) and those who had two adults living in a household (11.6% [stress], 13.6% [FMD], and 19.4% [depression]). 

Prevalences were significantly higher among those who identified as gay, lesbian, bisexual, and something other than straight than among those who identified as straight. The highest prevalences occurred among those who were bisexual (34.3% [stress], 40.5% [FMD], and 54.4% [depression]). Those who identified as gay had lower prevalences for stress (18.3%), FMD (20.8%), and depression (34.0%) than did those who identified as lesbian, bisexual, or something else. Prevalences were significantly higher among those who were transgender than among those who were cisgender, with the highest prevalence of depression (67.2%) occuring among those who were transgender gender nonconforming.

## Discussion

This analysis reinforces existing evidence that loneliness and lack of social and emotional support are associated with depression and stress (*7*). The findings in this report also identified that prevalences of loneliness, lack of social and emotional support, stress, FMD, and depression were significantly higher among bisexual and transgender persons than among heterosexual and cisgender persons. Among demographic categories, prevalences of loneliness and lack of social and emotional support were high in young adults, most racial and ethnic minority groups, and among those with less than a high school education and low income; these results are consistent with previous reports (*7*,*8*).

These findings highlight the importance of integrating standardized measures of social connection into existing data systems and public health frameworks and initiatives (*1*,*7*). Improved surveillance can identify risk factors and help guide interventions to address the impacts of loneliness and lack of social and emotional support. This study offers further evidence of the need to increase access to mental health and social services and address mental health outcomes related to loneliness and lack of social and emotional support, especially among SGM groups, who report the highest prevalence rates.

### Limitations

The findings in this report are subject to at least four limitations. First, BRFSS data are self-reported, which can result in recall and social desirability biases. Second, only 26 states reported data from both the Social Determinants and Health Equity module and the Sexual Orientation and Gender Identity module; therefore, results might not be generalizable to the entire U.S. adult population. Third, low response rates in some states (range = 36.9% [Delaware] to 59.6% [Alaska]) could result in nonresponse bias and missing data on household income, sexual orientation, gender identity variables, and stress might introduce information bias; however, the application of sampling weights helps address this bias. Finally, the 2022 BRFSS measured loneliness indirectly by asking persons whether they felt socially isolated, which could be misunderstood as a measure of social isolation. The wording was changed in the 2023 BRFSS to measure loneliness directly (i.e., “How often do you feel lonely?”).

### Implications for Public Health Practice

Evidence-based interventions and strategies that address social connection as a protective factor for mental health and well-being are needed, especially for persons who face disparities based on race, education, income, and SGM status. Developing environments in communities that are safe spaces for relationship building and support for dealing with loneliness and isolation can be beneficial (*4*).

Providing access to health services that are affirming for SGM groups and collecting data to address health inequities might help improve delivery of culturally competent care. The health care system, including hospital settings, outpatient clinics, emergency departments, and other health care settings, can play a role in raising awareness, promoting a welcoming environment, using gender-neutral and inclusive language, and reducing the stigma around loneliness (*9*). Worsening mental health among sexual and gender minority SGM populations suggests a need for mental health and primary care providers to address the unique psychosocial needs of these populations (*10*). Collecting data on SGM populations is also essential to providing high-quality, patient-centered care.^¶^ Lack of information could result in missed opportunities to identify specific health care needs of SGM populations, address the health disparities they experience, and deliver important health care services.

## Footnotes

* https://www.cdc.gov/brfss

† https://www.cdc.gov/brfss/annual_data/2022/pdf/Complex-Sampling-Weights-and-Preparing-Module-Data-for-Analysis-2022-508.pdf

§ 45 C.F.R. part 46.102(l)(2), 21 C.F.R. part 56; 42 U.S.C. Sect. 241(d); 5 U.S.C. Sect. 552a; 44 U.S.C. Sect. 3501 et seq.

¶ https://snapshot2024.cdc.gov/hiv/clinicians/transforming-health/health-care-providers/collecting-sexual-orientation.html
